# Advances in Chemistry and Bioactivity of Magnoflorine and Magnoflorine-Containing Extracts

**DOI:** 10.3390/ijms21041330

**Published:** 2020-02-16

**Authors:** Estera Okon, Wirginia Kukula-Koch, Agata Jarzab, Marta Halasa, Andrzej Stepulak, Anna Wawruszak

**Affiliations:** 1 Department of Biochemistry and Molecular Biology, Medical University of Lublin, Chodzki 1 St., 20-093 Lublin, Poland; estera.okon@umlub.pl (E.O.); agatajarzab@umlub.pl (A.J.); martahalasa@umlub.pl (M.H.); andrzejstepulak@umlub.pl (A.S.); 2 Department of Pharmacognosy, Medical University of Lublin, Chodzki 1 St., 20-093 Lublin, Poland

**Keywords:** magnoflorine, natural products, alkaloids, isoquinoline alkaloids, aporphine alkaloids, biological activity

## Abstract

The review collects together some recent information on the identity and pharmacological properties of magnoflorine, a quaternary aporphine alkaloid, that is widely distributed within the representatives of several botanical families like Berberidaceae, Magnoliaceae, Papaveraceae, or Menispermaceae. Several findings published in the scientific publications mention its application in the treatment of a wide spectrum of diseases including inflammatory ones, allergies, hypertension, osteoporosis, bacterial, viral and fungal infections, and some civilization diseases like cancer, obesity, diabetes, dementia, or depression. The pharmacokinetics and perspectives on its introduction to therapeutic strategies will also be discussed.

## 1. Introduction

Natural products that contain a nitrogen atom in their structure, generally called alkaloids, belong to the most active metabolites of natural origin that have been extensively used as toxins and drugs throughout the history. Among several classes of these compounds, which are derived from different aminoacids in their biochemical synthesis pathways, the isoquinoline alkaloids constitute the largest and the most differentiated group that has been divided into several classes [1]. Among them, magnoflorine has been of particular interest to the authors of this review.

Magnoflorine (MGN) can be perceived as an underestimated secondary metabolite, even if it is widely distributed among various plant representatives. However, several scientific manuscripts investigate the pharmacological potential of this alkaloid and determine its presence in both plant extracts and biological samples.

The aim of the review was to collect the pharmacological properties of MGN, which have been proven and described in the scientific manuscripts over the period of last three decades, and to draw the researchers’ attention to this molecule, which exhibits an interesting pharmacological potential.

## 2. Chemistry and Pharmacokinetics of Magnoflorine

**Figure 1:**
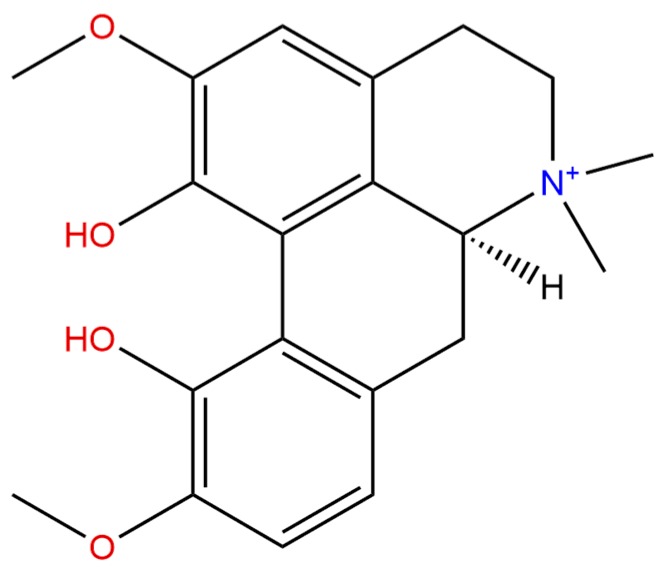
Chemical structure of (+)-(S)-magnoflorine (α-magnoflorine)**.**

MGN (Figure 1) from a chemical point of view belongs to isoquinoline alkaloids, and more precisely–to their aporphine derivatives. These compounds are derived from benzylisoquinolines in the process of two hydrogen atoms’ subtraction, which results in the formation of a 9,10-dihydrophenantrene structure out of the two benzene nuclei [2]. Aporphine alkaloids are widespread among the representatives of ca. 20 botanical families such as Papaveraceae, Berberidaceae, Annonaceae, Aristolochiaceae, Lauraceae, Monimiaceae, Menispermaceae, and others [2,3,4] (Table 1).

MGN itself can be perceived as the most widely distributed aporphine compound. Certainly, it is an interesting alkaloid characterized by the presence of two hydroxyl- and two methoxyl-substituents attached to an aporphine structure that occurs naturally in various plant species with distant etymology. Structurally, it is a direct derivative of (S)-corytuberine in which the nitrogen atom has been quaternized by another methyl group, and as the result of this process, MGN occurs in the plants in the form of a quarternary ammonium ion with high polarity and good water solubility [3,4,36,37]. From the phylogenetic point of view, MGN resembles the structure of aristolochic acid derivatives [7].

Scarce literature data describe the configurational isomerism or chirality of MGN of natural origin. According to the majority of authors, this compound is reported as (+)-(S)-magnoflorine [38,39,40]. However, the work of Chen and co-investigators [25] confirmed the presence of two isoforms of MGN that exhibit different pharmacological activity potential, which is further described in the Section 3.10.2. In their work, α-and β-magnoflorine were isolated from the aerial parts of *Clematis parviloba*. The optical rotations and melting point of both metabolites were determined as [α] _D_^26^ + 240.0 (c 0.1, MeOH), 197–198 °C for β –MGN and [α]_D_^26^ +150.0 (c 0.1, MeOH), and 243–244 °C for α –MGN, respectively. Based on the obtained NMR spectra, these two isoforms were assigned as R-form and S-form, respectively [25].

Other, better studied compounds that belong to the group of alkaloids have a clear isomerism profile defined in the former studies. The presence of enantiomers among plant-derived secondary metabolites indicates that they could possibly exhibit distinct therapeutic potential [41,42]. Among such compounds, (−)-quinine and its quasi-enantiomer (+)-quinidine can be mentioned. Their chirality markedly influences their pharmacological profile. It has been widely discussed that the former alkaloid exhibits strong antimalarial properties, whereas the latter—antiarrhythmic properties, and that this marked difference in activity is directly related to the structural differences. According to the current knowledge, the physiological impact of MGN isoforms needs further studies, but from the scarce literature data, it can be assumed that they tend to show a different pharmacological potential.

As a nitrogen-containing compound, MGN is easily detectable in the positive ionization mode when analyzed by mass spectrometers. A wide range of studies have been performed to confirm its presence and quantify it in various plant species, mainly using an LC-MS approach. The majority of studies have been performed using a standard chromatographic column filled with an RP-18 silica gel. MGN appears to be washed out of the column in the intermediate polarity conditions—around 20–40% of acetonitrile in water in the temperature range of 20–30 °C [8,43]. An addition of modifiers to the mobile phase is often described for isoquinoline-alkaloids-containing compounds [4,44,45]. Ammonium formate, ammonium acetate, formic acid, acetic acid, and ammonia solutions are the most often found solutions that increase the formation of sharp and even peaks of MGN, and reduce its elution time [8,46]. The UV spectra of MGN exhibit three maxima: for S-MGN, they were reported as 205, 227, and 275 nm, whereas for R-MGN—205, 227, and 273 nm [25].

The methoxyl and hydroxyl substituents of MGN are easily removable in the fragmentation process performed on mass spectrometers. This technique is certainly the technique of first choice in the precise determination of MGN in both plant and animal tissues. The parent ion of MGN is visible in the mass chromatograms as *m*/*z* of 342.17. The main fragment ions comprise the following signals: 311, 297, 282, 265, and 237 that are due to the presence of [M-MeO]^+^, [M-C_2_H_7_N]^+^, [M-C_2_H_7_N-Me]^+^, [M-C_2_H_7_N-MeOH]^+^, and [M-C_2_H_7_N-MeOH-CO]^+^ ions, respectively [4,45]. The analyses performed by LC-Q-TOF-MS instruments show a wide window of MGN detection—the fragmentation energies can range from 100–200 V to obtain clear spectra, whereas the collision energy values can exceed even 30 V to deliver sufficient intensity of signals suitable for its identification. The highest signal intensity was recorded for capillary voltage of 3000 V within the tested range of 3000–4000 V [8]. On the other hand, the analysis of MGN on triple-quadrupole mass spectrometers, e.g., in a study performed by Xia and colleagues, the following parameters were selected as optimal for the determination of the transition of precursor ion (342.1) to product ion (297.1) in plant and biological samples: fragmentor voltage: 100 V and collision energy: 10 eV [43]. The aim of the review was to collect the pharmacological properties of MGN, which have been proven and described in the scientific manuscripts over the period of last three decades, and to draw the researchers’ attention to this underestimated molecule, which exhibits an interesting pharmacological potential.

### Pharmacokinetics of Magnoflorine

There are just several reports on the bioavailability of MGN evaluated in animal models. In the study of Tang and collaborators [47], a daily intragastric administration of a complex preparation Xian-Ling-Gu-Bao used in traditional Chinese medicine was studied. Pharmacokinetics of MGN among other 20 components was evaluated in rats upon 1 g/kg/day oral administration. As a result, the bioavailability of MGN was determined as maximal after 0.54 ± 0.34 h, its half-time recorded as 5.68 ± 7.51 h, the maximal concentration as 38.16 ± 29.29 ng/mL, and the total exposure to drug expressed as an area under the curve as AUC0-t: 75.34 ± 42.68 and AUC0-∞ 85.74 ± 51.63 ng × h × mL^−1^. Its values of mean residence time were equal to: 2.72 ± 1.27 h MRT0-t and 5.63 ± 4.74 h for MRT0-∞. These data show that MGN has been immediately absorbed and reached high *Cmax* after oral administration.

The permeability and absorption of MGN after oral administration in rats was also studied by other authors investigating the pharmacokinetics of the same preparation. Jin and co-workers [48] treated the animals with 13.3 mL/kg of the preparation and studied the composition of the blood samples after 0.08, 0.17, 0.25, 0.5, 1, 2, 4, 6, 8, 12, 24, and 36 h. In their research, the maximum concentration of MGN was observed after 1.53 ± 1.46 h and was equal to 8.30 ± 2.06 ng/mL, with the half-time calculated as 11.62 ± 18.87 h. This particular study concluded that MGN was absorbed moderately, exhibited extremely low plasma concentration (*Cmax* lower that 10 ng/mL), and within a longer *Tmax* in relation to the first above described study.

The same alkaloid determined in rat plasma after the administration of ermiao pill that is composed of *Phellodendri cortex* and *Atractylodis rhizoma* showed marked differences in the bioavailability of several isoquinoline alkaloids present in these plants. MGN was the second best available alkaloid after berberine. Among other secondary metabolites that are commonly present in the plant extracts rich in MGN like palmatine, berberrubine, or epiberberine, whose bioavailability was very low, the reviewed alkaloid exhibits an almost 10-fold higher potential. However, its *Tmax* value was reached later than that of other compounds—after more than 2 h in relation to 1.7 h or even 1 h for other compounds [43]. The same authors have analyzed the detailed mechanism of action that the group of isoquinolines exhibits to explain their traditional usage in pelvic inflammatory disease. In comparison with other metabolites, MGN was the only compound targeting the *TLR4* and *MAPK8* genes in relation to berberine derivatives that affected *PTGS1*, *PTGS2, ESR1*, and *NOS2* genes.

The studies on the excretion kinetics of a MGN-containing Chinese traditional medicine preparation that has a *Coptis chinensis* extract were performed on urine and feces samples of healthy and insomniac rats by Chen and colleagues [49]. Research on the pharmacokinetic profile of MGN suggested that under pathological states, like a developed insomnia in the tested animals, the levels of pharmacodynamics features related to the excretion process were disturbed. After a 7-day-long oral administration of 3 g/kg/day of Jiao-Tai-Wan preparation, the urinary and fecal excretion of the preparation decreased in insomniac rats. Due to a longer presence of MGN in the bloodstream, according to the authors, its pharmacological effect was also prolonged.

Xue and colleagues [45] proved that there is a significant difference in the absorption of MGN in different parts of the digestive tracts. Also, the results differ when the alkaloid is administered alone or in the group (in the total extract of *C. chinensis*). In duodenum, the absorption of MGN was higher when present in the extract (1.41 ± 0.33 µg/cm^3^ in relation to 1.12 ± 0.32 µg/cm^3^ when alone); in colon, the absorption was similar for both types of administration (0.78 ± 0.12 µg/cm^3^ and 0.82 ± 0.13 µg/cm^3^), whereas, in the jejunum and ileum, MGN was better absorbed when alone (1.97 ± 0.25 and 1.31 ± 0.23 µg/cm^3^ for MGN alone, respectively, in contrast to 1.68 ± 0.60 and 1.11 ± 0.48 when in the form of an extract).

The same authors have studied the behavior of MGN in the digestive tract. These researchers have successfully identified several metabolites that arise from the biotransformation process, among them are monohydroxylated, mono-demethylated, di-demethylated, and mono-dehydrogenated derivatives of MGN. Additionally, some ketonization and glucosylation products, or glucuronidated metabolites were identified by this research group in the rat plasma [45].

## 3. Biological Activities

Recent studies have demonstrated that MGN shows a number of pharmacological effects including antidiabetic [50], antioxidant [51,52], anti-inflammatory [53], immunomodulatory [54,55], antiallergic, anticancer [47,56], cardiovascular (relaxant) [57], anti-osteoporotic, antibacterial [58], antifungal [25], or antiviral [59] activities. This compound has also been proven to cross the blood–brain barrier and exhibit central action—affecting the regulation of the central nervous system. The latter properties were proven in antidepressant [60] or antiamnestic [4,61] tests performed in animal models.

### 3.1. The Effect on Carbohydrate–Lipid Metabolism

Numerous plants have been widely used in the traditional medicine for the treatment of diabetes mellitus. *Tinospora cordifolia* or *C. chinensis* extracts are the sources of several active components, among which a group of isoquinoline alkaloids takes an important place [20,62,63]. MGN, which belongs to this group, together with jatrorrhizine, berberine, tembetarine, and hydrastine became the matter of attention because of its antidiabetic effects [50,55].

Diabetes mellitus can be an essential reason of blindness, kidney failure, cardiovascular disorders, or amputations among adults [64]. Progress of the disease leads to the development of macro- and microvascular complications associated with diabetes, which is the huge threat in a postprandial hyperglycemia [65]. There are several therapeutic protocols that use amylin analogs, α-glucosidase inhibitors, or aldose reductase inhibitors in the treatment of diabetic complications [66,67]. Aldose reductase is one of the important enzymes in the polyol pathway that is responsible for the reduction of glucose to sorbitol, the accumulation of which can cause retinopathy, neuropathy, nephropathy, and cataracts [68].

It was proved that *T. cordifolia* extract containing MGN shows aldose reductase inhibitory activity [67,69]. Moreover, MGN was found to inhibit α-glucosidase enzyme in vivo in rats in an oral glucose tolerance test with different substrates: glucose, sucrose, and maltose. It occurred that the plasma glucose level was significantly suppressed. Moreover, it was proven that MGN induced increase of K_m_, and due to this fact, it can be perceived as a reversible, competitive inhibitor of α-glucosidase. Additionally, the alkaloid was found to decrease glucose level in the oral glucose tolerance test in rats [67].

Other studies showed that the isoquinoline alkaloid rich fraction that was obtained from the stems of *T. cordifolia* significantly decreased gluconeogenesis in rat hepatocytes and increased insulin secretion in *Rattus norvegicus* RINm5F cell line. The influence of the isolated alkaloids on insulin secretion in the absence and presence of glucose (16.7 mM) was assessed. In the first case, MGN showed significant increase of insulin secretion after the administration of 20 µg/mL, whilst in the presence of 16.7 mM glucose, MGN significantly stimulated insulin secretion in a dose-dependent manner. Moreover, oral administration of various doses (50, 100, and 200 mg/kg) of alkaloid fraction as well as MGN individually (40 mg/kg each) reduced fasting serum glucose level and extinguished an increase of blood glucose level after 2 g/kg glucose loading in normal rats. Additionally, alkaloidal fraction from the same plant decreased gluconeogenesis in rat hepatocytes similar to insulin, and this mechanism stimulated the secretion of insulin in RINm5F rat pancreatic β-cell line like tolbutamide, an oral antihyperglycemic agent. This demonstrates that active fraction of alkaloids shows a hypoglycemic activity by mechanisms of insulin-releasing or insulin-mimicking action, thus ameliorating postprandial hyperglycemia [50].

It was also demonstrated that MGN had moderate glucose lowering activity. The combination of *Coptidis rhizoma* alkaloids showed synergistic effect on antihyperglycemic effect in liver hepatocellular carcinoma HepG2 cells in vitro [70].

*In vitro* screening of alkaloid-rich liver cell extract obtained after the administration of *Coptidis rhizoma* and a free fatty acid-induced test for hepatic steatosis HepG2 cell assay was performed by Fan and colleagues [71]. Hepatic steatosis characterized by overmuch fat accumulation in hepatocytes is a serious clinical problem. In the free fatty acid-induced test for hepatic steatosis, HepG2 cell assay Oil Red O staining and intracellular triglyceride contents were used to evaluate the activity of the compound on lowering the lipid levels within the cells. The tested extract at the concentration of 12.5 to 100.0 µg/mL had a triglycerides reducing effect in a dose-dependent manner. MGN and other alkaloids from the extract like epiberberine, palmatine, or columbamine reduced triglyceride level in a statistically significant way only at the highest concentration applied (5.0 µg/mL) [71].

Anti-diabetic potential of *Coptis* alkaloids, including MGN, was estimated by the evaluation of the ability of these active compounds to inhibit the protein tyrosine phosphatase 1B (PTP1B) and the ONOO (-)-mediated protein tyrosine nitration by Choi and co-investigators [72]. MGN showed significant inhibitory activity against PTP1B enzyme with the IC_50_ value of 28.14 µM, compared to the ursolic acid (positive control). Kinetic studies with the Lineweaver-Burk and Dixon plots revealed that MGN noncompetitively inhibits the PTP1B activity. The alkaloid also suppressed the ONOO (-)-mediated tyrosine nitration in a dose-dependent manner. These results clearly demonstrated the promising anti-diabetic potential of Coptis alkaloids, including MGN, and let them be treated as PTP1B enzyme inhibitors and ONOO (-)-mediated protein tyrosine nitration suppressors [72].

Additionally, the alkaloids from *C. chinensis* roots were evaluated to check their ability to promote the glucose uptake by skeletal muscles in C2C12 myotubes. To assess the effect of *C. chinensis* roots on glucose utilization in skeletal muscles, ethanol extract was prepared and partitioned with water, n-butanol, and dichloromethane. n-Butanol and dichloromethane sub-fractions stimulated glucose uptake in differentiated C2C12 cells at 50 μg/mL in the strongest way. These findings suggest that active constituents of *C. chinensis* root can reduce hyperglycaemia in diabetes by promoting glucose uptake in skeletal muscles [46].

#### Hypercholesterolemia and Obesity

Obesity is a multifactorial, chronic disease that increases the risk of diabetes type 2. As the disease is being diagnosed in an increasing number of patients, new anti-obesity and cholesterol lowering drugs are still being searched for. It was found that the alcoholic extracts from the rhizome of Coptis chinensis that contain MGN are able to significantly inhibit adipocyte differentiation and lipid contents in tests performed on *Mus musculus* 3T3-L1 cell line. In other scientific studies, it was also stated that five alkaloids isolated from Coptidis rhizoma extract inhibited lipids’ accumulation in vitro without affecting cell viability dependently on the concentration applied. Moreover, these alkaloids significantly inhibited adipogenesis in a dose-dependent manner, in vitro, as assessed by Oil-Red O staining and reduced the expression of some marker genes such as proliferator activated receptor-γ (PPAR-γ) and CCAT/enhancer binding protein-α (C/EBP-α). The influence of MGN isolated from the n-butanol fraction of *Coptidis rhizoma* on adipogenesis and cell viability was investigated by the Oil-Red O staining and MTT assay, respectively. To assess the inhibitory effect of MGN on adipocyte differentiation, 3T3-L1 preadipocytes were differentiated in the presence of different doses (12.5, 25, and 50 μM) of MGN. 3T3-L1 cells were fully differentiated during adipocyte differentiation. Accumulation of lipids was observed with microscopic and Oil-Red O staining. Interestingly, MGN (50 μM) inhibited the accumulation of cellular triglycerides in 3T3-L1 adipocytes at 38.87% and reduced the accumulation of lipids in the 3T3-L1 adipocytes in a dose-dependent manner with an IC_50_ value of 68.8 μM. All these results indicate that *Coptidis rhizoma* extract and its isolated alkaloids can be interesting potentially-active molecules in the treatment of obesity [73].

Other in vitro studies revealed that Jingi formula containing the extract from *C. chinensis*, *Lonicera japonica*, and *Astragalus membranaceus* also suppresses the accumulation of triglycerides and free fatty acids in mature *Mus musculus* embryo 3T3-L1 adipocytes through an increase in the expression and tyrosine phosphorylation of 5’-AMP-activated protein kinase (AMPK) and a decrease in the expression of lipid metabolism enzymes: acetyl-CoA carboxylase (ACC), hormone sensitive lipase (HSL), and fatty acid synthase (FAS). Furthermore, in vivo studies demonstrated that Jingi formula reduced body weight without changing food intake and decreased the concentration of serum glucose, triglycerides, and free fatty acids. Moreover, these extracts increased the expression and tyrosine phosphorylation of AMPK in the liver and muscles in mice. Finally, according to the studies, Jinqi formula decreased the expression of HSL and ACC and stimulated the expression of insulin receptor substrate 1 (IRS-1) in their livers. On the other hand, it decreased the expression of ACC, and increased the expression of glucose transporter type 4 (GLUT-4) and insulin receptor substrate 2 (IRS-2) [74].

Another traditional Korean medicine, Sam-Hwang-Sa-Sim-Tang (SHSST), composed of three herbs: *Coptidis rhizoma, Scutellariae radix,* and *Rhei rhizoma*, was also found to suppress the development of hyperlipidemia. SHSST diminished serum triglycerides (TC) and low-density lipoprotein (LDL) levels. It also lowered the sterol regulatory element-binding protein (SREBP), which is a key transcription protein in cholesterol metabolism. Moreover, it inhibited the hepatic mRNA expression of the liver X receptor (LXR), the low density lipoprotein receptor (LDLR), sterol regulatory element-binding protein 2 (SREBP-2), and β-hydroxy β-methylglutaryl-CoA (HMG-CoA) in vivo, which could suggest the protective properties against hepatic steatosis and atherosclerosis. The herbal extract administration showed the decrease in the level of total cholesterol and low-density protein in serum of mice. Histological examination showed that lipid droplets were smaller within the group after the extract’s administration. Additionally, the expression of SREBP-2 protein was diminished by this extract at the protein level, which was analyzed by Western blotting. Finally, the mRNA expression of molecules taking part in the cholesterol metabolism like SREBP-2, LXR, LDLR, and HMG-CoA were also suppressed after administration of SHSST extract as stated in real-time polymerase chain reaction analysis [60].

Coptidis rhizoma alcohol extract was also suggested to promote the conversion of cholesterol into bile acids by increasing cholesterol 7 alpha-hydroxylase (CYP7A1) activity in the liver of hyperlipidemic rats, which was measured in a colorimetric assay. The mechanism could also be related to the up-regulation of PPARα and the negative modulation of the nuclear farnesoid X receptor (FXR, bile acid receptor) in vivo, when analyzing the mRNA and protein expression by RT-PCR and Western blot, respectively [75].

The other studies indicated that *C. chinensis* alkaloids reduced the body weight gain and the total cholesterol, TG, LDL, total bile acids, and lipopolysaccharides levels in serum of hyperlipidemic B6 mice. These effects may testify that examined alkaloids are agonists of farnesoid X receptor (FXR) and G-protein-coupled bile acid receptor (TGR5, Gpbar1); activators for SREBP2, mitochondrial uncoupling protein (2UCP2) and CYP7A1; and inhibitors for 3-hydroxy-3-methylglutaryl-CoA reductase (HMGCR), thioredoxin-interacting protein (TXNIP), toll-like receptor 4 (TLR4), and c-Jun N-terminal kinase (JNK). Obesity, connected with an occurrence of chronic inflammatory response, demonstrates the progression of infection, the occurrence of TLR4 mediated inflammatory response, and the activation of JNK pathway by TLR signaling. It finally results in the insulin resistance. *Coptis* alkaloids in the performed tests affected JNK and TLR4 pathways by the modulation of gut microbiota [76].

Moreover, it has been shown that the levels of total cholesterol, LDL, and oxidized LDL, were significantly reduced in a dose-dependent manner in rats treated with *Coptidis rhizoma* extract orally at the doses of 50 and 100 mg/kg body weight/day during 30 days. The extract effectively reduced the pathological damage caused by hypercholesterolemia, by decreasing the serum cholesterol level. The studied extract also reduced the level of liver cholesterol but it did not affect the level of fecal cholesterol. Decrease in the cholesterol level occurs as a result of the reduction of cholesterol synthesis and not of the enhancement of its excretion. Moreover, *Coptis* extracts prevented hypercholesterolemic disease by reducing serum thiobarbituric acid-reactive substance levels and lipid peroxidation. All these studies suggest that isoquinoline alkaloids may be useful in the therapy of hypercholesterolemia by reducing cholesterol levels and oxidative stress [77].

### 3.2. Antioxidant Properties

Antioxidants are a group of compounds that are able to neutralize free radicals (FRs) and other reactive oxygen species (ROS). Disproportion between FR generation and antioxidant defenses results in oxidative stress leading to protein, lipid, and nucleic acid degradation. The oxidative stress is considered as a key player in inflammatory processes and cancer, as well as many other human disorders. Despite the fact that ROS exert destructive effect on normal cells, they are also essential for physiological balance inside the cell [78,79].

MGN isolated from *Mahonia aquifolium* seems to be a promising antioxidant due to the hydroxyl moieties present in its structure. This alkaloid exhibits both antioxidant and antiradical properties, which was revealed in a successful inhibition of lipid peroxydation and radical scavenging potential, respectively. To determinate the antioxidant activity of the compound, the dioleoyl phosphatidylcholine (DOPC) liposomes were used, and the properties were compared with stobadine and Trolox, as reference compounds. The liposomal membrane perooxidation was triggered in the thermal degradation of 2,2’-Azobis(2-amidinopropane) dihydrochloride (AAPH). As a result of this assay, the antioxidant activity of the tested compound was defined by the time and the percentage of inhibition of DOPC liposomes generated by MGN. Both tests showed similar effects and confirmed a comparable antioxidant potential of MGN with stobadine and a significantly higher potential from Trolox. Other tests that led to the determination of MGN’s antiradical potential were based on the measurement of the free 2,2-diphenyl-1-picrylhydrazyl radical (DPPH) stability (measured at 518 nm). The results showed that MGN was less reactive than the standards; however, its antioxidant potential was comparable with stobadine. The presence of −OH groups in the MGN’s chemical structure significantly influenced its oxidative properties through an easier donation of phenolic hydrogen, which in turn affects chain-breaking mechanism. In general, the free-radical scavenging properties enhance with an increase in the number of hydroxyl groups. MGN with the unsubstituted −OH groups scavenged free stable DPPH radical; however, it showed less activity compared with stobadine. On the other hand, antioxidative efficiency of MGN with dihydroxylated groups was comparable with standards [80].

To confirm that free-radical scavenging properties depend on the number and position of −OH groups, MGN isolated from *Epimedium elatum* was again tested in a DPPH assay. As it was mentioned above, DPPH has its characteristic absorption, which is significantly decreased after the exposition to radical scavengers through hydrogen or electron donation. The lower the absorbance value measured at 517 nm, the higher the radical scavenging activity of a sample. As expected, MGN showed scavenging effect in dose-dependent manner with an IC50 of 4.91 µM [81].

Furthermore, MGN isolated from *Coptidis rhizoma* had a significant inhibitory influence on high-density lipoprotein (HDL) oxidation. The level of HDL in blood is associated with coronary heart disease risk. The cardio-protective effect exerted by HDL could be connected with its antioxidative mechanism, wherein the low-density lipoprotein (LDL) is protected against oxidative modification. The HDL oxidation was determined by the conjugated dienes’ formation that caused the UV absorbance changes at 232 nm. HDL was pre-incubated with the presence of various concentrations of MGN and in its absence, which was used as a control, next Cu^2+^ was added as an oxidation indicator, and absorbance was continuously measured at 232 nm, and the lag time was determined. Due to the assumption that the formation of conjugated dienes is the initiation phase of HDL oxidation, the length of lag time is considered as HDL oxidation resistant capacity. The results indicated that the lag time for HDL incubated with Cu^2+^ alone was 62 min, while after MGN administration, the lag time was extended to 123 min. Even at low concentrations of MGN (3.0 mM), a protective effect against lipid peroxidation of HDL induced by Cu^2+^ was noted in a dose-dependent manner. The HDL oxidation initiated by Cu^2+^ to malondialdehyde (MDA) was also measured with the aid of the thiobarbituric acid reactive substances (TBARS). The results showed that MFN significantly reduced TBARS formation, wherein the results are comparable with conjugated diene formation. As a conclusion, the antioxidant effect toward HDL oxidation may be correlated with metal ion chelating properties of MGN [51,52].

Moreover, MGN isolated from *C. chinensis* rhizome inhibited the oxidation of both glycated and glycoxidated LDL induced by Cu^2+^, augmented lag time of conjugated dienes, and inhibited the production of TBARS. The oxidation initiated by Cu^2+^ toward various forms of LDL, including glycated and glycoxidated LDL, was determined by MDA formation. MGN showed antioxidative properties toward LDL and glycated and glycoxidated LDL oxidation with IC50 of 3.7, 4.3, and 6.5 µM, respectively. Additionally, the influence of MGN on the LDL lag-time change for the formation of conjugated dienes was investigated. The results showed that MGN at a concentration of 4 µM was able to prolong the lag phase from 38 min (LDL incubated with Cu^2+^ without MGN) to 64 min. The protective properties against apolipoprotein B (apo B) oxidative changes prompted by Cu^2+^ of MGN were also detected using a fluorescence assay. The administration of MGN increased the level of fluorescence in a dose-dependent manner. The antioxidative properties of MGN seem to be connected with the protection of apolipoprotein B (apoB) structural modification, which is essential for LDL oxidation [51].

Alkaloids isolated from *Coptis japonica* Makino are also characterized by their strong anti-photooxidative properties, which result from their chemical structure. The methanolic extracts of this plant were partitioned by Kim and colleagues [82] with butanol, ethyl acetate, and diethyl ether. The antioxidative properties of isolated fractions were investigated due to chlorophyll-sensitized photooxidation of linoleic acid. Chlorophyll is known as effective generator of singlet oxygen under light conditions. Thus, oxidation of linoleic acid could be initiated by singlet oxygen generated during chlorophyll and light presence. Nevertheless, the linoleic acid is able to initiate reaction of radical chain during the oxidation progress. The results indicated that antiphotooxidative properties of butanol fraction, that also contained MGN, were significantly higher compared with other fractions and exerted 52.1% prevention of photooxidation of linoleic acid [82].

### 3.3. Anti-Alzheimier’s and Anti-Aging Effect

The antioxidant potential of MGN isolated from *C. chinenis* rhizome has been evaluated in respect of therapeutic effect in Alzheimer’s disease (AD) [83]. The oxidative stress plays a prominent role in AD progression through the aggregation of amyloid β (Aβ), which generally accompanies an increased ROS production. Despite previous demonstration of the antioxidant properties of MGN, in this case, MGN did not show inhibitory effect of ROS within tested concentrations [84].

On the other hand, an in vivo study emphasized the positive effect of MGN on a short-term and long-term memory at a 20 mg/kg b.w. dose. After MGN administration, the cognitive processes of animals have been significantly enhanced, which could be proved by an extended time of the animals’ transition to the dark room during the applied passive avoidance test. The animals were able to recognize the correlation between dark room and negative electric stimulus, thus the time spent by mice in a bright room has been extended and the animals showed reluctance to enter the dark compartment. Importantly, only one dose of MGN increased the cognitive mechanism of animals, assessed after 2 h training session and after 24 h, which confirmed that MGN affects both short- and long-term memory. The half of the dose showed analogous pro-cognitive tendency, but the results were not statistically significant. Moreover, MGN had an influence on reversing the impairment of long-term memory induced by scopolamine in both doses when compared with a control group [4].

### 3.4. Anti-Inflammatory, Immunomodulatory, and Anti-Allergic Effects

Immune system is an interactive network involved in fundamental physiological processes including reproduction, wound healing, and development, and is correlated with other body systems such as metabolism, central nervous system, and cardiovascular system. Immune system is highly adaptive and possesses self-regulating and memory properties [85,86].

Currently, immunomodulation is considered as a key player in tissue homeostasis, leading to body physiological stability. Natural compounds significantly contribute to immunomodulatory therapies as they seem to show minimal side effects [87]. MGN isolated from *T. crispa* (TC) is considered as a potential immunomodulator due to its immunomodulatory effects confirmed in in vitro research. Among all alkaloids isolated from TC, MGN turned out to be the most active, significantly inducing migration and augmenting the phagocytic activity in mouse macrophages RAW 264.7 cell line. The better phagocyte function of macrophages induced by TC extract, the stronger increase of innate immune response was observed. Moreover, the MGN contributed to production of pro-inflammatory cytokines, including TNF-α, IL-1β, and IL-6 in RAW 264.7 macrophages in a dose-dependent manner. MGN also meaningfully stimulated production of prostaglandin E2 (PGE2) [88]. Interestingly, macrophage’s phagocytic potential after administration of MGN in 200 mg/kg dose was comparable with control group administrated with 2.5 mg/kg of kevamisole, and no significant differences were observed between MGN-administrated and control group. The results indicate that TC extract enhances the ability to phagocytose, induced by peritoneal macrophages. The activation of phagocytes in mice liver has been determined by activity of myeloperoxidase (MPO). The TC extract increased MPO activity in a dose-dependent manner, and reached a significant level at 200 mg/kg dose [89]. The effect of TC extract on humoral immune response was detected by established level of serum immunoglobulins (IgG and IgM). The mice with TC extract administration exhibited significantly higher level of IgG and IgM, wherein the maximum response was detected at 100 mg/kg dose. At this concentration, the immunoglobulins response was higher than in control group. Raising the dose of MGN up to 200 mg/kg caused a minor reduction of immunoglobins, but still the level of IgG and IgM remained meaningfully higher compared with control group [89]. This alkaloid was able to increase the pro-inflammatory cytokines production, including TNF-α and IL-1β protein expression, as well as mRNA expression in lipopolysaccharide (LPS)-activated macrophages in a dose-dependent manner. However, MGN did not affect the LPS-knockout macrophages [90]. MGN meaningfully upregulated the phosphorylation of p65—a subunit of NF-κB, wherein the total level of p65 remained unchanged. NF- κB plays a key role in an inflammatory response regulation and ensures a homeostasis of the immune system, simultaneously. The MGN’s effect has been examined on cyclooxygenase-2 (COX-2), which is known as an important pro-infammatory mediator, and its key product: (prostaglandin E_2_) PGE2. The experiment showed that expression of both proteins was significantly increased after MGN pre-treatment in LPS-activated U937 macrophages in a dose-dependent manner. [84]. Moreover, MGN promoted the phosphorylation and ubiquitynation of nuclear factor of kappa light polypeptide gene enhancer in B-cells inhibitor, alpha (IκBα), which is responsible for inhibition of NF-κB transcription activity. Consequently, MGN supported IKKα/β phosphorylation, which in turn took part in IκBα phosphorylation. MGN also exerted effect on Akt and MAPKs’ phosphorylation in LPS-primed U937 macrophages. MGN upregulated the Akt phosphorylation, and enhanced phosphorylation of JNK1/2, ERK11/2, and p38 MAPKs in LPS-activated macrophages in a dose-dependent manner [90]. MGN supported myeloid differentiation primary response gene 88 (MyD88) and toll-like receptor 4 (TLR4) activation [90]. MyD88 is known as an adaptor for inflammatory signaling pathways, and TLR4 recognizes LPS in LPS-mediated inflammatory response [91,92]. Taken together, MGN increases macrophage function through intensifying release of cytokines, which are known as pro-inflammatory markers, and modulation of MyD-88-dependent signaling events including NF-κB, MAPK, and Akt pathways in LPS-activated macrophages [90]. According to these findings, TC extract seems to be a strong immunostimulant, which owes its properties to alkaloids, mainly MGN. The anti-inflammatory properties of MGN have been tested in vivo in LPS-induced acute lung injury (ALI), and its potential molecular mechanism in RAW264.7 cells has been examined. Lung tissue damage has been analyzed via histopathological analysis and myeloperoxidase (MPO) assay on BALB/c male mice divided into few groups. Interestingly, pathological changes that occurred in the LPS group were observed, whereas the same pathological changes were significantly reduced in MGN-implemented groups. Furthermore, MPO test also indicated that LPS meaningfully increased the activity of myeloperoxidase, and this effect was reduced after MGN treatment [93].

The molecular effect of MGN has been determined by expression of inflammatory factors in lung tissue and RAW264.7 cell lines. In contrast to the above-described findings [90], the expression of IL-1β, IL-6, and TNF-α was dramatically higher in LPS group, compared with control group, whereas the expression of the same inflammatory factors has been reduced in MGN-implemented group in a dose-dependent manner. Moreover, MGN reduced TLR4 expression and p65 expression. Interestingly, MGN exerted an inhibitory effect on MAPK signaling pathway. The results showed that expression of proteins—phosphorylated p38, ERK, and JNK were lower after MGN treatment in a dose-dependent manner [90,93] contradicting previous findings. Additionally, MGN exhibited no cytotoxicity effect on RAW264.7 cells [94]. Other findings confirmed that the *Sinomenii caulis* extract, in which MGN is found, significantly reduced the NF-κB expression [55]. More specific, the MGN isolated from *Sinomenii caulis* presented significant inhibitory effect on NF-κB and inflammatory cytokines, including IL-6 and IL-8, whose transcription is affected by NF-κB [94].

MGN isolated from *Coptidis rhizoma* has been evaluated for its inhibition of CYP450 isoforms [95]. CYP450 enzymes are responsible for metabolism of many drugs and xenobiotics. Their polymorphism of CYP450 enzyme is essential in differentiation in response to medications due to the ethnicity of the patients [96,97]. This alkaloid did not show inhibitory effect of each tested CYP450 isoform in concentration up to 100 µM [95]. The in vivo research for *Caulophyllum robustum* Maxim extract indicates that MGN in connection with N-methylcytisine, saponins, sapogenins, and β-sitosterols (all compounds contained in this extract) showed an anti-inflammatory effect. However, the saponins were significantly better correlated with inflammatory factors than alkaloids [98].

### 3.5. Anticancer Activity

MGN promotes anti-proliferative effect of doxorubicin (DOX) in breast cancer cells but it does not affect normal cells. DOX shows high anticancer activity but its use is limited due to its cardiac toxicity. Approximately 60% of DOX-treated patients develop cardiomyopathy. Viability of MCF7, MDA-MB-231, MDA-MB-453, and BT474 breast cancer cells was strongly reduced after MGN treatment in the concentration range 10–80 μM. Interestingly, MGN did not affect MCF-10A human normal mammary epithelial cell line. DOX decreased MCF-7 and MDA-MB-231 cell viability, which was promoted through the addition of MGN in a dose-dependent manner. Isobolographic analyses showed that a combination of DOX and MGN generates a synergistic effect in MCF7 and MDA-MB-231 cancer cells. Moreover, MGN promoted anti-migration and anti-invasion effects of DOX in breast cancer cells. MGN used individually, as opposed to DOX, did not inhibit migration and invasion. The DOX effect was enhanced after the administration of both compounds in combination. MGN and DOX applied together increased expression of epithelial marker E-cadherin and decreased mesenchymal N-cadherin, vimentin, and α-smooth muscle actin (α-SMA) mRNA level compared to DOX separately. Flow cytometry results revealed that MCF-7 and MDA-MB-231 cells accumulated largely in the G2/M phase after DOX treatment. This effect was promoted by MGN co-treatment. DOX reduced the expression of cyclin B1, cyclin-dependent kinase 1 (CDK1), and cyclin-dependent kinase 2 (CDK2), which was further decreased after DOX/MGN co-treatment. Moreover, DOX/MGN treatment induced apoptosis compared to DOX individually by the reduction of B-cell lymphoma 2 (Bcl-2) expression and enhancement of caspase-3 and -9 cleavage. DOX/MGN induced a higher percentage of autophagic cells than that in the DOX group, which accompanied up-regulation of Beclin-1 and LC3-II expression. p62 expression was reduced, as well as p21 and p53 protein levels were induced after DOX/MGN treatment, compared to DOX alone. mCherry-GFP-LC3 experiments revealed that DOX/MGN can significantly up-regulate the numbers of GFP-mCherry +(red) puncta, which demonstrates an increase in autolysosomes, compared to the DOX group. The results of these experiments clearly suggest that MGN promotes anti-cancer effect of DOX by inducing cellular apoptosis and autophagy in breast cancer cells. Additionally, co-treatment of DOX with MGN strongly inhibited the activation of phosphatidylinositol 3-kinase/protein kinase B/mammalian target of rapamycin (PI3K/AKT/mTOR) signaling and promoted p38 mitogen-activated protein kinase (MAPK) pathway. Moreover, treatment with a blocker of autophagosome formation (wortmannin) significantly reduced DOX/MGN-induced p38 MAPK activation and LC3 conversion in human breast cancer cells.

DOX/MGN also strongly reduced the tumor growth in vivo. DOX and MGN displayed anti-tumor effect in MCF7 xenograft model with relatively low toxicity to heart, liver, kidney, and spleen. The tumor growth rate was slower in DOX-treated group, which was further promoted in DOX plus MGN group along with a significant reduction in the change of tumor weight. No weight loss and correct blood indexes in mice after DOX/MGN treatment suggested that the DOX/MGN in combination show few side effects in vivo. Western blotting analysis revealed that expression of p53, LC3-II, cleaved Caspase-3, and p-p38 was induced, and phospho-AKT (p-AKT), phospho-PI3K (p-PI3K), and phospho-mTOR (p-mTOR) expression was significantly downregulated by DOX/MGN combinational treatment.

In summary, the combination of MGN with DOX significantly inhibited proliferation, migration, and invasion of breast cancer cells, and induced apoptosis through mitochondria-dependent pathway and cell distribution in G2/M phases. Moreover, MGN/DOX treatment resulted in the activation of autophagy via LC3-II regulated through p38 and PI3K/AKT signaling pathways in breast cancer. MGN strongly enhanced DNA damage and sensitivity of breast cancer cells to DOX treatment [56].

It has been demonstrated that MGN can be an important antitumor agent. MGN obtained from fractionation of the methanol extract from *Magnolia grandiflora* leaves inhibited cell viability of the Hela cervix tumor cell line, U251 brain tumor cell line, and HEPG2 hepatocellular carcinoma cell line. IC_50_ 0.4 mg/mL of MGN against HEPG2 was only two times higher than IC_50_ of standard cytostatic—doxorubacin (IC_50_ 0.27 mg/mL). Moreover, MGN exhibited cytotoxicity against U251 cell line (IC_50_ = 7 µg/mL), but it was inactive relative to Hela cancer cells [59].

In turn, another research group demonstrated that MGN isolated from fruit of *Ziziphus jujube* shows a very weak, statistically insignificant cytotoxic effect against MCF7 breast cancer, A549 lung cancer, HepG2 hepatocellular carcinoma, and HT-29 colon cancer cell lines [32].

Studies have demonstrated that not only MGN in the form of a pure compound but also the extracts of which it is a component show anti-cancer activity.

The influence of *Coptidis rhizoma* aqueous extract (CRAE) containing 2.2% of MGN, 4.4% palmatine, and 13.8% berberine on vascular endothelial growth factor (VEGF) expression and angiogenesis in hepatocellular carcinoma (HCC) has been investigated. CRAE exerted 50% of cytotoxicity against MHCC97L and HEP G2 cells after 48 h at the doses of 150 μg/mL and 120 μg/mL, respectively. Therefore, the nontoxic doses of 75 and 150 μg/mL of CRAE were used in subsequent experiments. Results from ELISA test showed more than two-fold secretory VEGF protein reduction in MHCC97L and HepG2 cells after 24 h’ treatment with CRAE. CRAE inhibits VEGF protein expression independent of its cytotoxicity. Unexpectedly, an increase in the expression of VEGF mRNA was noticed. All these results suggest that CRAE does not affect VEGF transcriptional level but rather VEGF protein secretion. CRAE inhibited secretion and synthesis of VEGF protein but not mRNA transcript. Moreover, CRAE treatment increased the phosphorylation of eukaryotic elongation factor 2 (eEF2), inhibiting VEGF synthesis in MHCC97L and Hep G2 cells. It indicates that blockade of VEGF synthesis is associated with inactivation of eEF2 and disruption of the translation process in HCC cells after CRAE treatment. Reduction of neovascularization level and tumor size were observed in mice xenograft model. Histologic analysis with the use of Ki-67 antibody demonstrated a significant decrease in areas of proliferating cells in mice compared to control after CRAE treatment. The presence of CD31-postive cells also decreased in CRAE-treated mice, demonstrating the reduced rate of blood vessel formation. Moreover, CRAE-treated mice showed a lower vascular density in tumor compared to the control group.

Despite the fact that berberine is the main component of CRAE, the whole extract was more active than berberine alone, suggesting an additive effect of other extract ingredients. Therefore, CRAE may be considered as a potential anti-angiogenic remedy for HCC [99].

*T. cordifolia* extract, fractions, and eight molecules including MGN have been isolated, characterized based on NMR and mass spectroscopy, and evaluated for the anti-cancer activity against four different types of cancer cells including KB human oral squamous carcinoma, HT-29 human colon cancer, CHOK-1 hamster ovary, and SiHa human cervical cancer, as well as murine primary cells. The pharmacological assessment of extract, selected fractions, and pure molecules exposed the ethnomedicinal importance of *T. cordifolia* for immunomodulatory and anticancer activities. The highest cytotoxicity against KB and CHOK-1 cells showed ethyl acetate fraction (TCEF-03) and parent extract (TCEWF-01) with IC_50_ value of 52.7 μg/mL and 18.5 μg/mL, respectively. Based on the evaluation of the extract, individual fractions, and pure substances, it has been noticed that the activity of the whole extract was greater than activity of fractions and individual substances. It reveals that the activity is not concentrated to only one particular molecule but rather distributed to multiple molecules, which show a synergistic effect [100].

### 3.6. Depressant Effect on the Central Nervous System

The central depressant actions of MGN, as an active agent of methanol extract from *Coptis* root, were tested in mice. It has been shown that coordinative motor activity and spontaneous movement were not depressed by all methanol extracts and MGN fraction, as well as other extract fractions such as non-alkaloid fraction quarternary base fraction, berberine, and coptisine hydrochlorides. There was no sign of inhibition of electro- and chemical-shock-induced convulsion, Straub’s tail reaction induced by morphine, apomorphine-induced masticating motion, and electrically stimulated aggressive behavior [61].

### 3.7. Antidepressant Effect

The results of the forced swim test (FST) and the tail suspension test (TST) in the chronic unpredictable mild stress animal model demonstrated that MGN–phospholipid complex has significant antidepressant effect. MGN and MGN–phospholipid complex groups were compared with control model group in the FST. The results of the experiment showed that mice FST immobility time was significantly shorter after both MGN and MGN-complex treatment compared to control; however, MGN-complex was more effective than MGN in its original form at the same doses. Similarly, in TST, immobility time was significantly shorter after MGN and MGN-complex treatment compared to control, but the effects caused by MGN and MGN-complex were similar in the same doses. Additionally, MGN-complex prolonged the period of MGN in the blood and helped MGN to permeate the blood–brain barrier into the brain. All these findings firmly suggest that the MGN–phospholipid complex significantly improves antidepressant effect, drug properties, and liposolubility of MGN [60].

### 3.8. Antiosteoporosis Effect

MGN isolated from *Epimedii herba* in concentration 1.54–30.74 µg/mL is the component of the Xian-Ling-Gu-Bao (XLGB) capsule. XLGB capsule is an extensively used remedy in Chinese natural medicine for the treatment of osteoporosis [101]. It has been reported that XLGB shows anti-osteoporosis activity in ovariectomized mice [102,103] and rats with oral dosages at 236 mg/kg/day and 270 mg/kg/day, respectively [47]. Moreover, the dose up to 1800 mg/kg/day showed no serious side effects in ovariectomized rats. [47,103]. Dose 0.3 g/kg/day used for experiments was based on the body weight conversion to the clinical effective dose of XLGB used for the prevention of postmenopausal osteoporosis. The study also assessed the effectiveness and a positive effect on the bone health of XLGB in the prevention of osteoporosis in the above-mentioned rats at a dose of 1 g/kg/day. Simultaneous assessment of bioactive components in the plasma of rat after oral administration of XLGB at pharmacodynamic doses was efficiently achieved. Among them, MGN was considered as the key-effective substance of XLGB capsule due to their appropriate pharmacokinetic features and high exposure [47].

### 3.9. Cardiovascular Effects

The effect of MGN was tested on the isolated aorta from rat. MGN shows weak relaxation effect in noradrenaline- and KCl-induced contractions. However, MGN does not inhibit the phenylephrine concentration-response curve (CRC) [57].

Cardiac electropharmacological action of the Mokuboi-to was investigated. Mokuboi-to is a traditional herbal drug used for centuries in Japan. The major component of Mokuboi-to is *Sinomeni caulis et rhizome* (SCR) or *Sinomenium acutum* Rehder et Wilson, which contains *inter alia* MGN. Modulation of the action potentials and underlying ionic currents using patchclamp techniques were assessed. It has been shown that MGN at concentration 1 mM prolonged APD75 (action potential duration measured at 75% repolarization) in ventricular cardiomyocytes, but the effect was weaker than the effect caused by other Mokuboi-to compounds. MGN does not affect APA (action potential amplitude). Moreover, all the ionic currents were also unaffected. MGN had also weak or no effect on the action potential parameters in the papillary muscles [104].

In summary, MGN has very little or no effect on the cardiovascular system.

### 3.10. Anti-Microbial Activity

The plant extracts and active compounds isolated from these plants are generally available, non-hazardous, biodegradable, and sometimes show broad-spectrum activities against numerous microorganisms [105]. Studies have shown that MGN or plant extracts containing it in their composition have significant activity against different microorganisms, including bacteria [106], fungi [25], and viruses [59].

#### 3.10.1. Antibacterial Activity

In vivo studies demonstrate that the herbal components of *Coptidis rhizoma*, including MGN, [106], as well as *Mume fructus* and *Schizandrae fructus* have antibacterial activity against enterohemorrhagic *E. coli*, including strains resistant to many antibiotics. In in vivo experiments using antibacterial activity assay, the extract from *Coptidis rhizoma*, *Mume fructus*, and *Schizandrae fructus* was administered to mice after initial *E. coli* infection. It has been demonstrated that the extract from these herbal combination, decreased the release of Shiga toxin from EHEC O26, EHEC O111, and EHEC O157 strains assessed by the reversed passive latex agglutination method [58].

It has been shown that the same methanolic extract had antimicrobial activity toward 26 tested *Salmonella* strains. Some of these strains were resistant to multiple antibiotics. *S. gallinarum* infected chicken model was used for checking in vivo antibacterial activity of the extract. Animals treated with extract did not show any serious clinical signs and sparsely showed histological damage related to the disease. While untreated animals showed different clinical signs, inter alia congestion, and necrotic changes in the kidney, liver, and spleen. All these results suggest that the herbal combination composed of *Coptidis rhizoma, Mume fructus*, and *Schizandrae fructus* can be an effective treatment for salmonellosis [107].

Effect of *Coptidis rhizoma* on *Staphylococcus aureus* growth was assessed by microcalorimetry. Low concentration of extract from *C. chinensis* showed poor inhibitory effect, whereas a high concentration significantly inhibited the growth of this bacterium [108]. It has been shown using the ultra-performance liquid chromatography with photodiode array detector (UPLC-PAD) that antibacterial activity of the tested samples depends on their cultivated origins [109].

#### 3.10.2. Anti-Fungal Activity

Two isoforms of MGN such as MGN-α and -β isolated from the aerial parts of *Clematis parviloba* were found to have the antifungal activity against *Penicillium avellaneum* UC-4376. The minimal inhibition amounts of MGN-α against *P. avellaneum* UC-4376 was 10 μg/disc and against *P. oryzae* at 100 μg/disc. While the minimal inhibition amount of manoflorine-β against *P. avellaneum* UC-4376 was 5 μg/disc, no inhibitory activity against *P. oryzae* were noticed at 100 μg/disc [25].

MGN was found to have the highest growth inhibitory activity against *Candida* strains among 44 tested compounds, with a minimum inhibitory concentration of 50 μg/mL in the microdilution antifungal susceptibility testing. The antifungal activity of MGN was stable over 3 days as compared to those of berberine and cinnamaldehyde. At 50 μg/mL concentration, MGN inhibited 55.91% activity of alpha-glucosidase, which is required for virulence and cell wall composition of *Candida albicans*. Moreover, MGN decreased the formation of *C. albicans’* biofilm. Interestingly, a combined treatment of miconazole with MGN reduced the concentration of miconazole required to kill *Candida albicans*. Moreover, MGN did not show toxicity against spontaneously transformed aneuploid immortal keratinocyte HaCaT cell line from adult human skin. All these findings suggest that MGN is a promising candidate for novel antifungal agents [110].

Aqueous and ethanol extracts from *C. chinensis* rhizome and other Traditional Chinese medicines showed the significant antifungal properties against dermatophyte strains. The minimum inhibitory concentration (MIC) range of ethanol extracts from *Coptidis rhizoma* against dermatophyte strains were 0.156–1.250 mg/mL, with mean being 0.313 mg/mL. The minimum fungicidal concentration (MFC) range of ethanol extract from the plant was 1.250 ≥ ~2.500 mg/mL, with mean being 2.500 mg/mL [111].

#### 3.10.3. Antiviral Activity

The methanol extract of *Magnolia grandiflora* (Magnoliaceae) leaves containing MGN was found to exhibit high antiviral activity against herpes simplex virus (HSV-1) (76.7% inhibition at 1.1 mg/mL) and moderate antiviral activity against poliovirus type-1 (PV1) (47% inhibition at 1.1 mg/mL). Poliovirus type-1 as a model of an RNA virus and HSV-1 as a model of a DNA virus were used for antiviral bioassay (plaque reduction assay). Fractionation of the extract led to the isolation and characterization of four aporphine alkaloids MGN, liriodenine, lanuginosine, and anonaine [59].

Below, pharmacological properties of MGN and its effects on the metabolic pathways are presented, which confirm a multitude of its applications in the therapy of different disorders. Table 2 lists the major ones.

## 4. Perspectives

A plentiful of studies on the application of magnoflorine present in the extracts rich in isoquinoline alkaloids, or as an isolated compound, show an urgent need for further studies on this interesting metabolite.

The alkaloid has been traditionally used in phytotherapy and administered in the form of extracts (mainly decoctions) obtained from the plants like *Coptis chinensis*, *Tinospora cordifolia*, *Berberis vulgaris*, or others, so is the mixture of compounds that treated bacterial, fungal, viral, or protozoal infections, or increased bile production and digestion.

Together with the development of instrumentation and the availability of more precise and comprehensive techniques of plant metabolites’ identification, isolation, and bioactivity evaluation, new perspectives appeared in the case of magnoflorine application.

So far, several mechanisms of action of the reviewed metabolite have been proposed; however, more studies, especially those that include in vivo models on animals and on human with the application of a purified compound are needed to confirm the final pharmacokinetics and toxicity of this aporphine alkaloids’ representative. In addition, the structural issues should be clarified in the nearest future to exclude an eventual presence of other chiral or stereo-isomers of this compound in the natural sources. Other forms of MGN could have a crucial meaning in the bioactivity assessment or toxicity studies.

Certainly, this underestimated alkaloid will be of interest to many researchers, whose studies will confirm a significant impact of magnoflorine on a living organism.

## Figures and Tables

**Table 1:** The distribution of magnoflorine among plant species.

Botanical Family	Gender Name	Selected Species	References
**Annonaceae**	*Annona*	*A. glabra*	[5]
	*Asimina*	*A. triloba*	[5]
	*Enantia*	*E. chlorantha*	[6]
**Aristolochiaceae**	*Aristolochia*	*A. contoria*	[7]
**Berberidaceae**	*Berberis*	*B. vulgaris*, *B. cretica*, *B. siberica*	[4,8]
	*Caulophyllum*	*C. robustum*	[9]
	*Mahonia*	*M. napaulensis*, *M. leschenaultia*	[10,11]
	*Epimedium*	*E. grandiflorum*	[12]
	*Nandina*	*N. domestica*	[13]
**Menispermaceae**	*Cissampelos*	*C. pareira*	[14]
	*Sinomenium*	*S. acutum*	[15]
	*Stephania*	*S. kwangsinensis*; *S. glabra*	[16,17]
**Papaveraceae**	*Argemone*	*A. mexicana*, *A. grandiflora*	[4,8]
	*Corydalis*	*C. hendersonii,*	[18]
	*Glaucium*	*G. flavum*	[19]
	*Papaver*	*P. orientale*, *P. rhoeas*, *P. nudicaule*, *P. crocetum*	[8]
**Ranunculacae**	*Coptis*	*C. chinensis*	[20,21]
	*Adonis*	*A. aestivalis*	[22]
	*Nigella*	*N. sativa*, *N. glandulifera*	[23]
	*Helleborus*	*H. viridis*	[23]
	*Aquilegia*	*A. fragrans*	[24]
	*Clematis*	*C. recta*, *C. parviloba*	[23,25]
	*Thalictrum*	*T. foetidum*	[26]
	*Tinospora*	*T. crispa*, *T. sinensis*, *T. cordifolia*	[27]
	*Zanthoxylum*	*Z. nitidum*	[28]
**Rutaceae**	*Phellodendron*	*P. chinense*, *P. amurensis*	[29,30]
	*Toddalia*	*T. asiatica*	[31]
**Rhamnaceae**	*Ziziphus*	*Z spinosa*	[32]
**Euphorbiaceae**	*Croton*	*C. urucurana*	[33]
**Magnoliaceae**	*Magnolia*	*M. officinalis*	[34]
**Olacaceae**	*Ptychopetalum*	*P. olacoides*	[35]

**Table 2:** Biological activities of magnoflorine (MGN) and extracts containing magnoflorine.

Activity	Pure Substance/Extract	Model	Mechanism of Action	References
**The Effect on Carbohydrate–Lipid Metabolism**	*Tinospora cordifolia* extract	In vitro in rats and sheep lenses	Inhibition of aldose reductase activity.	[67,70]
	MGN	In vivo in rats	Inhibition of α- glucosidase competitive activity.	[67]
	Isoquinoline alkaloid rich fraction from the stem of *T. cordifolia*	In vivo in rat hepatocytes and in vitro in *Rattus norvegicus* RINm5F cell line	Decrease gluconeogenesis in rat hepatocytes and increase insulin secretion in *Rattus norvegicus* RINm5F cell line.	[50]
	Alkaloid extract from *Coptidis rhizoma*	In vitro in hepatic steatosis HepG2 cell	Reducing effect of triglycerides.	[71]
	MGN	In vitro enzyme kinetics and in silico molecular docking simulation	Inhibition of activity of PTP1B and ONOO (−)-mediated protein tyrosine nitration.	[72]
	Alkaloids from *Coptis chinensis* roots (N-butanol and dichloromethane sub-fractions)	Skeletal muscles in C2C12 myotubes	Reduction of hyperglycemia in diabetes through promoting glucose uptake in skeletal muscles.	[46]
	Methanol extract from *Coptidis rhizoma* containing MGN	In vitro in *Mus musculus* 3T3-L1 cell line	Inhibition of adipocyte differentiation and lipid contents in *Mus musculus* 3T3-L1 cell line.	[73]
	Alkaloid isolated from *Coptidis rhizoma* extract	In vitro in *Mus musculus* 3T3-L1 cell line	Reduction of the lipid accumulation, adipogenesis and expression ofPPAR-γ and C/EBP-α.	[73]
	MGN isolated from the n-butanol fraction of *Coptidis rhizoma*	In vitro in *Mus musculus* 3T3-L1 cell line	Inhibition of accumulation of cellular triglyceride and reduction of accumulation of the lipid in the 3T3-L1 adipocytes.	[73]
	Jingi formula containing the extract from *Coptidis rhizoma, Lonicerae japonicae*, and *Astragali radix*	Mature *Mus musculus* embryo 3T3-L1 adipocytes	Suppression of the accumulation of triglycerides and free fatty acids increase the expression and tyrosine phosphorylation of AMPK and decrease the expression of enzymes from lipid metabolism: ACC, HSL, and FAS.	[74]
	Jingi formula containing the extract from *Coptidis rhizoma, Lonicerae japonicae*, and *Astragali radix*	In vivo in mice	Body weight reduction without changing food intake and concentration of serum glucose, triglycerides, and free fatty acids. Increase of expression and tyrosine phosphorylation of AMPK. Reduction of the expression of HSL and ACC and stimulation of the expression of IRS-1 in mice livers.	[74]
	Sam-Hwang-Sa-Sim-Tang (SHSST), composed of three herbs: *Coptidis rhizoma, Scutellariae radix*, and *Rhei rhizoma*	In vivo in mice	Suppression of the development of hyperlipidemia. Reduction of the level of serum TC, LDL, SREBP, SREBP-2, LXR, LDLR, and HMG-CoA expression.	[112]
	Alcohol extract from *Coptidis rhizoma*	In vivo in rats	Promotion of the conversion of cholesterol into bile acids by increasing CYP7A1 activity, positive regulation of PPARα, and the negative modulation of the nuclear FXR, bile acid receptor.	[75]
	Alkaloids isolated from *Coptidis rhizoma*	In vivo hyperlipidemic B6 mice	Reduction of the body weight gain, total cholesterol, TG, LDL, total bile acids, and LPS in serum.	[76]
	Extract from *Coptidis rhizoma*	In vivo in rats	Reduction of the levels of total cholesterol, LDL, oxidized LDL, pathological damage caused by hypercholesterolemia, serum TBARS level, and lipid peroxidation.	[77]
**The Antioxidant Activity**	MGN isolated from *Mahonia aquifolium*	In vitro using DPPH test (antiradical scavenging activity) and AAPH test (antioxidant activity)	Ability to scavenge free stable DPPH radical and inhibition of lipid peroxidation.	[80]
	MGN isolated from *Epimedium elatum*	In vitro using DPPH assay	Ability to scavenge free stable DPPH radical.	[81]
	MGN isolated from *Coptidis rhizoma*	In vitro using AAPH and TBARS assay	Inhibition of oxidation high-density lipoprotein HDL exposed to Cu^2+^-independent form and peroxyl radicals, reduction of TBARS formation.	[52]
	MGN isolated from *Coptidis rhizoma*	In vitro using TBARS assay	Inhibition of oxidation of glycated and glycoxidated LDL induced by Cu2+ and prevention of production of TBARS.	[51]
	Alkaloids from *Coptis japonica*	In vitro using chlorophyll-sensitized photooxidation of linoleic acid.	Prevention of photooxidation of linoleic acid by butanol fraction	[82]
**The Anti-Alzheimer Activity**	MGN isolated from *Berberis cretica* root	In vivo study in mice	Raise in the cognitive processes of short-term and long-term memory.	[4]
**Anti-Inflammatory Activity**	MGN isolated from *Tinospora crispa*	In vitro, mouse macrophages RAW 264.7 cell line	Contribution to the production of pro-inflammatory cytokines (TNF-α, IL-1β, and IL-6) and stimulation of the PGE2production.	[88]
	Extract from *T. crispa*	In vivo study in mice	Establishment in the level of serum immunoglobulins (IgG and IgM).	[89]
	MGN isolated from *T. crispa*	In vitro, in LPS-activated U937 macrophages	Upregulation of the Phosphorylation of p65 and increase of COX-2 and PGE2 expression, promotion the phosphorylation, and ubiquitination of IκBα, upregulation of the phosphorylation of p65 and increase of COX-2 and PGE2 expression, promotion of the phosphorylation and ubiquitination of IκBα, upregulation of the Akt phosphorylation, enhancement of the phosphorylation of JNK1/2, ERK11/2, and p38 MAPKs, support in MyD88 and TLR4 activation.	[90]
	MGN isolated from *Sinomenii caulis*	In vivo study in mice and guinea pigs	Inhibition of NF- κB and inflammatory cytokines (IL-6 and IL-8).	[94]
	MGN	In vivo study in LPS-induced ALI in mice	Reduction of pathological changes induced by LPS.	[93]
**Anticancer Activity**	MGN	In vitro in MCF7, MDA-MB-231, MDA-MB-453, and BT474 breast cancer cells	Promotion of anti-cancer effect of DOX by inducing cellular apoptosis and autophagy in breast cancer cells. Reduction of viability, proliferation, migration, and invasion of breast cancer cells as well as increase expression of epithelial marker E-cadherin and decreased of mesenchymal N-cadherin, vimentin, and α-SMA after MGN and DOX treatment compared to DOX separately.	[56]
	MGN/DOX	In vivo in MCF7 xenograft model	Reduction of the tumor growth. Expression of p53, LC3-II, cleaved Caspase-3, and induction of phospho-p38, phospho-AKT, and phospho-PI3K and downregulation of phospho-mTOR expression after DOX/MGN combinational treatment.	[56]
	MGN from the methanol extract of *Magnolia grandiflora* L. leaves	In vivo in Hela cervix tumor cell line, U251 brain tumor cell line and HEPG2 hepatocellular carcinoma cell line	Inhibition of cell viability and cytotoxixity.	[59]
	MGN isolated from *Ziziphus jujube* fruit	In vivo in MCF7 breast cancer, A549 lung cancer, HepG2 hepatocellular carcinoma, and HT-29 colon cancer cell lines	Weak cytotoxic effect.	[32]
	*Coptidis rhizoma* aqueous extract	In vivo in MHCC97L and HEP G2 hepatocellular carcinoma cells	Cytotoxic effect; reduction of VEGF protein secretion, inactivation of the elongation factor 2 EF2.	[99]
	Water extract from *Coptidis rhizoma*	In vivo in mice model	Reduction of neovascularization level and tumor size.	[99]
	*T. cordifolia* extract	KB human oral squamous carcinoma, HT-29 human colon cancer, CHOK-1 hamster ovary, and SiHa human cervical cancer	Cytotoxicity effect.	[100]
**Effect on the Nervous System**	Methanol extract from *Coptis* root	In vivo in mice	No activity demonstrated.	[61]
**Antidepressant Effect**	MGN and MGN-phospholipid complex	Chronic unpredictable mild stress animal model	Significant improvement in the antidepressant effect, drug properties, and liposolubility of MGN induced by MGN–phospholipid complex.	[60]
**Antiosteoporosis Effect**	Xian-Ling-Gu-Bao (XLGB) capsule containing MGN	In vivo in ovariectomized mice and rats	Positive effect on the bone health.	[47]
	MGN	Isolated aorta from rat	Weak relaxation effect in noradrenaline- and KCl-induced contractions.	[57]
**Cardiovascular Effects**	MGN isolated from Mokuboi-to drug	Ventricular cardimyocytes from Guinea Pig heart	Prolongation of the APD75; weak or no effect on the action potential parameters in the papillary muscles.	[104]
**Antibacterial Activity**	Methanolic extract isolated from herbal combination containing MGN	In vivo in mice after initial enterohemorrhagic *E. coli* infection (EHEC)	Reduction of the release of Shiga toxin from EHEC O26, EHEC O111, and EHEC O157 strains.	[58]
	Methanolic extract isolated from herbal combination containing MGN	*S. Gallinarum* infection chicken model	Reduction of the clinical signs *inter alia* congestion and necrotic changes in the kidney, liver, and spleen.	[107]
	Extract from *R. coptidis*	In vitro in *Staphylococcus aureus*	Inhibition of the growth of bacteria.	[108]
**Anti-Fungal Activity**	MGN-α and -β isolated from the aerial parts of *Clematis parviloba*	In vitro *Penicillium avellaneum* and *Penicillium oryzae*	Inhibition of the growth of *Penicillium avellaneum* and *Penicillium oryzae.*	[25]
	MGN	*Candida* strains	Inhibition of the activity of α-glucosidase, which is required for virulence and cell-wall composition of *Candida albicans*. Reduction of the formation of *C. albicans’* biofilm.	[110]
	Aqueous and ethanol extracts isolated from *Coptidis rhizoma*	Dermatophyte strains	Inhibition of the growth.	[111]
**Antiviral Activity**	The methanol extract isolated form *Magnnolia grandiflora*	In vitro HSV-1 and PV1	Antiviral activity in a plaque reduction bioassay.	[59]

AAPH, 2,2’-Azobis(2-amidinopropane) dihydrochloride; ACC, acetyl-CoA carboxylase; Akt, Protein kinase B; ALI, acute lung injury; AMPK, 5’ AMP-activated protein kinase; APD75, action potential duration measured at 75% repolarization; COX-2, cyclooxygenase-2; CYP7A1, cholesterol 7 alpha-hydroxylase; C/EBP, α-CCAT/enhancer binding protein-α; DOX, doxorubicin; DPPH, 2,2-diphenyl-1-picrylhydrazyl; EF2, elongation factor 2; ERK, extracellular signal-regulated kinase; FAS, fatty acid synthase; FAR, farnesoid X receptor; HMG-CoA, 3-hydroxy-3-methyl-glutaryl-coenzyme A reductase; HDL, high-density lipoprotein; HSL, hormone sensitive lipase; HSV, herpes simplex virus; IgG, immunoglobulin G; IgM, immunoglobulin M; IL-1β, interleukin 1 beta; IL-6, interleukin 6; IL-8, interleukin 8; NF-κB, nuclear factor kappa-light-chain-enhancer of activated B cells; IκBα, nuclear factor of kappa light polypeptide gene enhancer in B-cells inhibitor; IRS-1, insulin receptor substrate 1; JNK, c-jun N-terminal kinase; LC3, microtubule-associated proteins 1A/1B light chain 3B; LDL, low-density lipoprotein; LDLR, low-density lipoprotein receptor; LXR, liver X receptor; LPS, lipopolysaccharides; MAPK, mitogen-activated protein kinase; *MyD88*, myeloid differentiation primary response gene 88; m-TOR, mammalian target of rapamycin; NF-κB, nuclear factor kappa-light-chain-enhancer of activated B cells; ONOO(-), peroxynitrite; PGE_2_, prostaglandin E_2_; PI3K, phosphoinositide 3-kinase; PPARα, proliferator activated receptor-α; PPAR-γ, proliferator activated receptor-γ; PTP1B, protein tyrosine phosphatase 1B; PV1, poliovirus type-1; SREBP, sterol regulatory element-binding protein; SREBP-2, sterol regulatory element-binding protein 2; TBARS, thiobarbituric acid reactive substances; TC, triglycerides; TNF-α, tumor necrosis factor alpha; TRL, toll-like receptor; VEGF, vascular endothelial growth factor; α-SMA, α-Smooth muscle actin.
